# Cars, CONSORT 2010, and Clinical Practice

**DOI:** 10.1186/1745-6215-11-33

**Published:** 2010-03-24

**Authors:** Hywel C Williams

**Affiliations:** 1 Centre of Evidence-Based Dermatology, University of Nottingham, Nottingham, NG7 2UH, UK

## Abstract

Just like you would not buy a car without key information such as service history, you would not "buy" a clinical trial report without key information such as concealment of allocation. Implementation of the updated CONSORT 2010 statement enables the reader to see exactly what was done in a trial, to whom and when. A fully "CONSORTed" trial report does not necessarily mean the trial is a good one, but at least the reader can make a judgement. Clear reporting is a pre-requisite for judgement of study quality. The CONSORT statement evolves as empirical research moves on. CONSORT 2010 is even clearer than before and includes some new items with a particular emphasis on selective reporting of outcomes. The challenge is for everyone to use it.

## Introduction

I have bought many cars in my time. I search on the Internet for specific models, and I make sure that they have some key attributes such as a full service history, road tax, certificate of roadworthiness and vehicle registration document before I spend time viewing them. That does not mean to say that the "full service history" is a good history, or that the registration document is a genuine one, but at least the items are available for me to make a judgement. Like me, I suspect that you would not buy a car without such key information, so why should it be any different when we "buy" a randomized controlled trial report that does not mention certain essential features such as a description of randomization, allocation concealment, masking and whether all those randomized were included in the analysis?

When I started off in clinical research some twenty years ago, reporting key items in clinical trials was the exception rather than the rule, especially in my own field of dermatology [1]. But all that has changed or is changing now [2] thanks to endeavours such as the Consolidated Reporting of Trials (CONSORT) statement that lists essential items to be included in any trial report. I do not need to explain what the CONSORT checklist is all about to readers of *Trials*, but I do need to highlight the most recent update called CONSORT 2010 [3] published in *Trials*, and what it might mean for you and me.

## Discussion

### Quality of reporting is not the same as trial quality

First, it is worth emphasising that the CONSORT 2010 statement is not an instrument to judge the quality of a randomized control trial. As illustrated in Table 1, four categories are formed when the two domains of trial quality and trial reporting are combined into a four-celled matrix. One is a clearly reported well-designed trial that is useful for informing clinical practice. The other is a clearly reported flawed study - not ideal, but at least anyone can see the study shortcomings and make a judgement about such a report. The remaining two cells are the problems - the well-designed or flawed study that is poorly reported. Both are of limited utility because they lie in the grey zone of uncertainty. They may be sparkling diamonds in a pile of rubble or just rubble, and they may be difficult to distinguish from each other - an observation that illustrates the primacy of clear reporting.

**Table 1:** Trial reporting and study quality may be related but are not necessarily the same. All poorly reported trials reside in the grey zone of uncertainty

Reporting quality	Study quality	
	**Good**	**Flawed**
**Clear**	May be helpful for clinical practice.	At least you can tell it is flawed and make a judgment on utility.
**Poor**	A sparkling diamond -- but how do you know?	Difficult to distinguish from a good but poorly reported study.

### Evolutionary not revolutionary

It is also important to realize that deciding on a minimum list of essential items for clinical trial reports is a challenging task, based on a combination of common sense consensus and new evidence from empirical research. Because opinions and research change with time, so too the CONSORT statement evolves with time - initially in 2001 and now in 2010. General and specific changes to CONSORT 2010 are explained fully in the statement, but it is especially pleasing to note that the CONSORT Group have resisted the temptation to simply add more items. The Group have striven to make the interpretation of some older items even clearer e.g. by disaggregating items that were composed of multiple items. New specific items which excite me most include: a sub-item which asks author to explain any changes to the methods after the study starts; a better description of the interventions so that they can be replicated; a more detailed description of allocation concealment mechanisms rather than banal "get out of jail free" statements like "allocation was concealed"; a clearer description of exactly what was meant by intention to treat; and a requirement that the interpretation of the study findings should be consistent with the results. The updated statement now also includes an item which requires authors to say where the trial has been registered and whether a protocol is publicly available - essential aspects that allow referees and readers to check on whether the authors have done what they said they would do.

### Throwing darts

Perhaps the most important change for me is the emphasis on selective reporting of outcomes, which to me is like throwing a dart then drawing a dartboard around where it lands. There is now overwhelming evidence that selective reporting of outcomes occurs [4]. I see selective reporting of outcomes constantly in dermatology [5], especially when refereeing submitted articles. Sometimes it is due to naivety, sometimes it is due to financial conflicts of interest, and sometimes it is due to a misguided desire to "save face" by trying to make something positive out of a negative or inconclusive result. Some call it avoidable waste [6]. I call it a distorting the scientific record. Whilst some of the better dermatology journals have striven to adopt CONSORT, many of the remaining 240 specialist dermatology journals where "negative" trials end up (trials that are often critical when assembling the totality of evidence in systematic reviews) have not, reinforcing the inverse research reporting law - where the need for good trial reporting is most, the quality of reporting is least. Helping CONSORT 2010 to reach all the biomedical journals is a challenge that needs to be taken up by all of us.

### Influencing clinical research from trial design to clinic

CONSORT 2010 has the potential to play a critical role in influencing clinical research and clinical practice in a number of ways. First, I do believe that the statement has and will continue to influence better trial design. In my role of helping others to design clinical trials at our Nottingham Clinical Trials Unit, I find myself giving colleagues a hard time as a result of CONSORT to ensure that the procedures for concealing allocation are robust e.g. by ensuring that data on potentially eligible trial participants are entered irrevocably onto a database before remote randomisation allocates the treatment group. I also believe that CONSORT has led to better research commissioning. The NIHR Health Technology Assessment Board, which I now work for, insists that trial applications include a draft CONSORT flow chart of how participants will be identified and allocated. When it comes to trial publication, there is little doubt that CONSORT can improve the quality of reporting when adhered to, although not all journals have the resources to ensure such adherence. In my work as a systematic reviewer, it is such a joy to come across a clearly reported trial when abstracting data. Recent evidence has pointed to the importance of how failure to account for reporting outcome bias can lead to an exaggeration of effects and different conclusions in systematic reviews [7], so the emphasis on outcome reporting in CONSORT 2010 is timely. Finally, as a practicing clinician, it is so much easier to read trials that follow CONSORT in order to see exactly what was done, by whom and when.

## Conclusion

The benefits of CONSORT are manifest right from trial conception to the application of evidence to patients in the clinic. A trial that is "CONSORTED" gives a signal to the reader that they can find what they want to find. CONSORT 2010 is not a tool to catch out well intentioned researchers with a straightjacket of prescriptive reporting formats - it is simply an aid to ensure that a trial report contains key information. Whether you are buying a car or a trial report, you need essential information to help you decide whether it is a good one. CONSORT 2010 helps you to do that. Use CONSORT 2010 if you are a generator of research. Insist on it if you are a user of research.

## Competing interests

The author declares that they have no competing interests.
